# Ginseng-Angelica-Sansheng-Pulvis Boosts Neurogenesis Against Focal Cerebral Ischemia-Induced Neurological Deficiency

**DOI:** 10.3389/fnins.2019.00515

**Published:** 2019-05-29

**Authors:** Bowen Liu, Qian Zhang, Chienchih Ke, Zhenyan Xia, Cheng Luo, Yang Li, Xiaowei Guan, Xiang Cao, Yun Xu, Yonghua Zhao

**Affiliations:** ^1^Department of Neurology, Nanjing Drum Tower Hospital Clinical College of Traditional Chinese and Western Medicine, Nanjing University of Chinese Medicine, Nanjing, China; ^2^Department of Biotherapy, Shenzhen Luohu People’s Hospital, Shenzhen, China; ^3^Department of Medical Imaging and Radiological Sciences, Kaohsiung Medical University, Kaohsiung City, Taiwan; ^4^Biomedical Imaging Research Center, National Yang-Ming University, Taipei, Taiwan; ^5^State Key Laboratory of Quality Research in Chinese Medicine, Faculty of Chinese Medicine, Macau University of Science and Technology, Macau, China; ^6^Department of Human Anatomy and Histoembryology, School of Medicine and Life Sciences, Nanjing University of Chinese Medicine, Nanjing, China; ^7^Department of Neurology, Drum Tower Hospital, Medical School and The State Key Laboratory of Pharmaceutical Biotechnology, Institute of Brain Science, Nanjing University, Nanjing, China; ^8^State Key Laboratory of Quality Research in Chinese Medicine, Institute of Chinese Medical Sciences, University of Macau, Macau, China

**Keywords:** Ginseng-Angelica-Sansheng-Pulvis, ischemic stroke, neurogenesis, neurological deficiency, neural differentiation

## Abstract

**Background:**

The traditional Chinese medicine Ginseng-Angelica-Shanseng-Pulvis (GASP) has been used to treat stroke for 300 years. This present study investigated if it can induce increases in neurogenesis following cerebral ischemic injury.

**Methods:**

Rats following middle cerebral artery occlusion were orally treated with high, medium, and low doses of a standardized GASP extract.

**Results:**

After 14 days, treatment with GASP improved regional blood flow and infarction volume by magnetic resonance imaging scanning, enhanced Ki67^+^ expression in the subventricular zone, increased brain-derived neurotrophic factor (BDNF) secretion, Nestin, and bone morphogenetic protein (BMP) 2/4 expressions in the hippocampus in a dose-dependent manner. Interestingly, low-dose treatment with GASP downregulated doublecortin and Notch1 expressions in the hippocampus, as well as upregulated glial fibrillary acidic protein expression in the subgranular zone and hairy and enhancer of split (Hes) 5 expression in the hippocampus, while treatment with middle and high doses of GASP reversed these results. Meanwhile, the consumed time was shortened in the basket test and the adhesive removal test and the spending time on exploring novel objects was prolonged by GASP treatment whose effects were more obvious at day 14 post-ischemia.

**Conclusion:**

Our study demonstrates that treatment with GASP increases neurogenesis and ameliorates sensorimotor functions and recognition memory. We hypothesize that these effects are thought be mediated by an effect on the BMP2/4 pathway and Notch1/Hes5 signal. Due to its beneficial efficacy, GASP can be recognized as an alternative therapeutic agent for ischemic stroke.

## Introduction

Cerebrovascular disease seriously threatens human life and health, especially in middle-aged and elderly people. Ischemic stroke has been one of the most common cerebrovascular diseases, with high rates of morbidity and mortality (Kim and Johnston, 2013). The pathology of ischemic stroke is extremely complex, and as a result, there have been few significant advances in the efficacy of treatment options (Roth and Liesz, 2016). Recently, developments have suggested that the regenerative capacity of neural cells following ischemia is highly correlated with functional recovery (Zhang et al., 2001; Thored et al., 2006; Quentin et al., 2015).

Neurogenesis refers to the differentiation of neural stem cells (NSCs) into various types of neural cells, such as neurons and astrocytes. Neurogenesis occurs throughout physiological and pathological progresses. To date, two “reservoirs” of endogenous stem cells have been found in the brain: the subventricular zone (SVZ) of the lateral ventricles and the subgranular zone (SGZ) of the dentate gyrus in the hippocampus, which is important for learning and memory in the brain. Proliferated and differentiated NSCs in the two classical neurogenic niches result in stroke-induced neurogenesis (Quentin et al., 2015). Neurogenesis normally occurs post-stroke, and this is associated with the potential for functional improvement and rehabilitation. In stroke-induced neurogenesis in human ischemic penumbra, newborn neurons take precedence over localizing near blood vessels, where it contributes to establishing a microenvironmental corridor (Jin et al., 2006). Therapeutic outcomes against ischemia are dependent on the period of adult neurogenesis occurring in the SVZ and SGZ. Proliferated cells in the SVZ directly migrate toward the infarction zone and then differentiate and integrate with cerebral parenchyma in adult rodents after stroke (Thored et al., 2006; Yamashita et al., 2006), and then, the stress of middle cerebral artery occlusion (MCAo) stimulates the proliferation of neuronal progenitor cells in the SGZ (Yagita et al., 2001). These studies have demonstrated that maximizing the efficacy of the brain’s endogenous mechanisms of self-repair is a promising treatment approach.

Neuroepithelial cells highly express Nestin, which can be recognized as a marker of progenitor cells of both neuronal and glial lineage (Halbach, 2011), while doublecortin (DCX) represents the existence of neuroblasts and immature neurons (Quentin et al., 2015). Reactive astrocyte-like cells marked by glial fibrillary acidic protein (GFAP) are a widespread endogenous stem cellular potential source following brain injury (Robel et al., 2011). A population of GFAP^+^/Nestin^+^ radial cells in the SGZ generates self-renewing non-radial type 2 progenitors in turn to give rise to DCX^+^ neuroblast differentiation, indicating a possible reciprocal lineage relationship between DCX and GFAP/Nestin-positive cells (Aimone et al., 2014). In response to the stroke stress, many cytokines such as brain-derived neurotrophic factor (BDNF), vascular endothelial growth factor (VEGF), insulin-like growth factor-1, and fibroblast growth factor-2 participate in proliferation of progenitor cells in both the SVZ and SGZ (Wiltrout et al., 2007). Among them, BDNF is well-known as the key neurotrophic factor not only for neurogenesis but also for assisting in activity-dependent plasticity, i.e., how the various synapses link together after brain injury. It is a potent regulator of neural systems as a whole (Scharfman et al., 2005). Notch1 and bone morphogenetic protein (BMP) signaling pathways are considered as two major regulated approaches of NSCs in neurogenic niches (Jing et al., 2009; Ables et al., 2010). The helix-loop-helix gene Hes5 is an essential downstream target gene for Notch signaling, which regulates the maintenance and expansion of NSCs (Ohtsuka et al., 2001), and further downregulation of Hes5 expression induces cellular differentiation in NSC pool (Chiba, 2006).

Ginseng-Angelica-Sansheng-Pulvis (GASP) has been employed as a traditional Chinese treatment for stroke since the early Qing dynasty and is composed of *Panax ginseng* C.A. Mey., root and rhizome; *Angelica sinensis* (Oliv). Diels, root, and rhizome; as well as *Cinnamomum cassia* (L). J.Presl, stem bark. The herbal plants have been used as traditional medicines for 1000s of years in oriental countries and are considered to have the ability of rejuvenating the body. Studies suggest that total saponins derived from ginseng improve neurological deficits after focal cerebral ischemia by the enhancement of neurogenesis (Zheng et al., 2011). Previously, we investigated therapeutic effects of two active constituents of *A. sinensis*: sodium ferulate (SF) and *n*-butylidenephthalate (BP) on stroke in rats, and found that SF could increase the expression of Nestin in the ischemic infarction zone (Zhao et al., 2012). Moreover, combination of SF and BP further increased the expressions of VEGF, BDNF, and neuron-specific class III beta-tubulin in ischemic penumbra and reduced the infarction volume (Zhang et al., 2016). Trans-cinnamaldehyde, an essential component in *C. cassia*, has a potential neuroprotective effect against ischemic stroke (Chen et al., 2016). Our previous study has demonstrated that GASP extraction is an angiogenic switch, which contributes to reductions of infarction volume and mortality post-stroke (Liu et al., 2018). However, whether GASP extraction can boost neurogenesis after cerebral ischemia is still unknown. In this study, we investigate the efficacy and mechanism of GASP on neurogenesis and hypothesize that BMP2 and 4 and Notch1/Hes5 signal pathways are involved in the procedure of neurogenesis after ischemic stroke.

## Materials and Methods

### The Quality Control of Ginseng-Angelica-Shanseng-Pulvis

*Panax ginseng* C.A.Mey., root and rhizome (bath no. 160101); *A. sinensis* (Oliv). Diels, root, and rhizome (bath no. 160301); and *C. cassia* (L). J.Presl, stem bark (bath no. 160101) were obtained from Guangzhou Zisun Pharmaceutical, Co., Ltd., China. The herbal medicines were identified by Professor Quan Zhu and accordant with the standards of Chinese Pharmacopoeia (2015). After the procedure of steam distillation, the aqueous extract was mixed with the volatile oil and consequently was manufactured into GASP extraction. The detailed extracted method of GASP and its representative compounds including ferulic acid, cinnamaldehyde, ligustilide, and ginsenoside Rb1 by high-performance liquid chromatography (HPLC) analysis have been reported in our previous article (Liu et al., 2018). Comparison of the retention times with standard compounds (originated from National Institutes for Food and Drug Control, China) and production of main active compounds from volatile oil and (or) GASP extraction are listed in Table 1.

**Table 1:** Major compounds in the volatile oil and (or) Ginseng-Angelica- Shanseng-Pulvis (GASP) extraction.

Compound	*t*_R_ (min)	Yield (mg/g)
Ferulic acid	32.46	11.7 ± 0.3 (GASP)
Cinnamaldehyde	57.34	215.2 ± 0.2 (volatile oil) 6.5 ± 0.4 (GASP)
Ligustilide	81.71	172.4 ± 0.3 (volatile oil)
Ginsenoside Rb1	90.15	20.0 ± 0.4 (GASP)

### Permanent Middle Cerebral Artery Occlusion Model and Experimental Groups

Male Sprague–Dawley (SD) rats were purchased from the Laboratory Animal Service Centre of Chinese University of Hong Kong. This study was carried out in accordance with the principles of the NIH Guide for the Care and Use of Laboratory Animals (National Institutes of Health Publication No. 80-23, revised 1996) and recommendations of Animals in Neuroscience Research by the Society for Neuroscience, Institutional Animal Care and Use Committee of Macau University of Science and Technology. The protocol was approved by the Institutional Animal Care and Use Committee of Macau University of Science and Technology. The survey procedure of the MCAo model, enrollment criteria, and laboratory environmental conditions were consistent with previous study (Liu et al., 2018). Briefly, SD rats were anesthetized; subsequently, 4-0 surgical nylon suture coated with polylysine was inserted into the lumen of the right common carotid and advanced into the internal carotid artery until the origin of the middle cerebral artery was occluded, and sham-operated rats underwent the same surgery but without nylon suture inserted.

Forty-eight MCAo rats whose neurological deficit scores were at least two by using a score following the protocol of Menzies et al. (1992) were randomly divided into four groups: the MCAo group, and the low-, middle-, and high-dose GASP groups (12 rats per group). At the same time, six Sham-operated rats were recruited for a Sham group. Three dosages of GASP extraction (2.3, 4.6, and 9.2 g/kg) were dissolved into distilled water and prepared for three kinds of suspension as before. Rats in the low, middle, and high dosage groups were subjected to intragastric administration with three kinds of suspension twice a day at 7:00 and 19:00 for 3 days before and 14 days after MCAo. Simultaneously, rats in the MCAo group and Sham group were given the same volume of distilled water.

### Behavioral Tests

**FIGURE 1:**
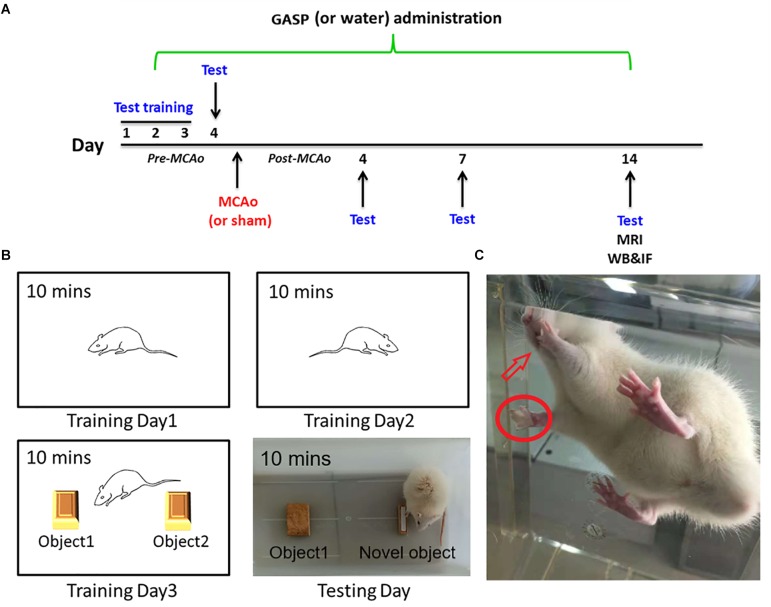
**(A)** The overall experimental process timeline. **(B)** The schematic of the novel object recognition test. **(C)** Rat in adhesive removal testing: the rat finds paper type sticking on its forelimb and tries to remove them using its tooth.

All rats were handled and trained five times every day, and it continued 3 days prior to the beginning of subsequent tests. The day before operation, behavioral tests were conducted, and continued at days 4, 7, and 14 after surgery by a blind-experimental operator (Figure 1A).

#### Basket Test

It is used to assess motor coordination and sensorimotor deficits in rodent models of central nervous system disorders, and its content originates from http://med.stanford.edu/sbfnl/services/bm/sm/BasketTest.html. Briefly, one rat was placed in the center of the open basket, which was made of horizontal and vertical cross wires with about 2 cm^2^ gap spacing. Subsequently, the operator inverted the basket. Rats were allowed to climb down the walls of the wire basket into their home cage. The time rats spent on completing the task was recorded, and each test was repeated three times.

#### Adhesive Removal Test

The sensitive method by Bouet et al. (2009) is used to evaluate sensorimotor deficits. Briefly, two small pieces of paper tape (of equal size square, 1 cm^2^) were taken as bilateral tactile stimuli to stick to the palmar surface of each forelimb (Figure 1C). The rats were then placed in their cages, and the operator observed the time on which the rats spent removing each tape by their teeth. Before surgery, all rats were able to remove the paper tape within 5 s at the end of training. The time required from the three repeated trials to remove both stimuli from each limb was recorded, and the mean time was calculated.

### Novel Object Recognition

The novel object recognition test is applied for the evaluation of cognition, particularly recognition memory in rodent models of central nervous system disorders. This test is based on the spontaneous tendency of rodents to spend more time on exploring a novel object rather than a familiar one, which reflects the actions of learning and recognition memory (Huang et al., 2014). The test comprised three parts and was performed for 4 days. The first part was being habituated. On the first and second training days, rats were put into a white box (40 cm × 40 cm × 30 cm) for 10 min to habituate to the manipulations and the environment. On the third training day, rats were put into the same box with two identical objects for 10 min to explore and recognize the objects. At testing day, the final memory test was performed. Rats were individually put into the box with an familiar object and one novel object for 10 min (Figure 1B). The test was conducted in a quiet environment, and the movements of rats in the box were recorded with a video camera. The ratio of time spent on the novel object was analyzed.

### T2W and DSC Examinations by Magnetic Resonance Imaging

Prior to imaging, rats anesthetized with an initial inhalation of 4% isoflurane for 3 min and maintained with 2% isoflurane in a mixture of 20% oxygen and 80% room air were placed in the stereotaxic holder of an MRI machine equipped with a heating system to maintain body temperature and a pressure detector to monitor respiration. At day 14, a Bruker Pharmascan 7-Tesla MR scanner (Bruker Biopsin MRI GmbH, Ettlingen, Germany) using a birdcage head coil of 75 mm inner diameter for radio frequency (RF) transmission and a 20-mm-diameter surface coil for reception was used for MRI scanning. MRI data sets consisting of T2W and DSC were acquired at corresponding time points. A Turbo Rapid Acquisition with Refocused Echoes (RARE) T_2_-weighted scan was used from Bruker BioSpin MRI GmbH: repetition time (TR) = 3,000 ms, effective echo time (TE) = 40 ms, rare factor = 4, number of average = 4; field of view (FOV) = 20 mm × 20 mm, slice thickness = 1 mm, matrix = 256 × 256, scan time = 6.24 min. DSC-MRI data were acquired with GE EPI with TE = 20 ms, TR = 1,000 ms, bandwidth = 250 kHz, and no averaging. Sixteen contiguous (1 mm thick) slices were recorded in axial orientation with an FOV of 20 mm × 20 mm and a matrix of 96 × 96 to give a nominal resolution of 208 μm × 208 μm. A series of 300 images with a temporal resolution of 1,000 ms were acquired. Eight coronal and eight horizontal slices were acquired covering a volume extending 10 mm in the rostrocaudal direction in both T2W and DSC. Coronal slices were centered around the infarction lesion, whereas horizontal slices were aligned with the skullcap.

### Immunofluorescence Staining

Rats (Sham group, *n* = 3, other groups *n* = 4) were sacrificed on the 14th day, and their brains were fixed in fresh 4% PFA. Fresh frozen coronal sections of 8 μm thickness were cut by a cryostat microtome (Shandon Cryotome FSE; Thermo Fischer Scientific). A total of six sections were used for immunofluorescence staining in the same position of every rat. To investigate cellular proliferation in the SVZ as well as neurogenesis in the SGZ, SVZ sections were incubated with antibody against Ki67 (Rabbit, 1:1,000; Abcam); three sections of the SGZ were incubated with DCX (Rabbit, 1:2,000; Abcam) overnight at 4°; then, samples were washed with PBS and incubated with the Alexa Fluor^®^ 488 goat anti-rabbit immunoglobulin (1:200; Life Technologies) for 1 h. After that, sections were incubated with NeuN (Mouse, 1:3,000; Abcam) in the same condition as DCX and Alexa Fluor^®^ 594 goat anti-mouse immunoglobulin (1:200; Life Technologies) secondary antibodies for 1 h at room temperature. The other three sections of the SGZ with GFAP (Rabbit, 1:2,000; Abcam) and Nestin (Mouse, 1:2,000; Abcam) staining were manipulated in the same way. Nuclei were stained with 4′, 6-diamidino-2-phenylindole (DAPI; Sigma). Fluorescent labeling was examined with the Leica TCS SP8 laser scanning confocal microscope (Leica Microsystems, Inc., Buffalo Grove, IL, United States). The immunofluorescence positive signals of each slice was evaluated by an investigator who was blind to the experiment under a magnification of 10 × 20 in confocal images. Image-Pro Plus software (Media Cybernetics, Rockville, MD, United States) was applied to measure the fluorescent positive density. In control experiments, primary antibodies were replaced with PBS.

### Western Blot

Protein samples were extracted from the hippocampus of each group (*n* = 3) after 14 days of administration. Protein concentration was determined by enhanced bicinchoninic acid (BCA) protein assay kit (Beyotime Institute of Biotechnology, Shanghai, China). Protein samples were electrophoresed on gradient sodium dodecyl sulfate (SDS)–polyacrylamide gel (Bio-Rad) and subsequently electrotransferred to polyvinylidene difluoride (PVDF) membranes (Bio-Rad) in Tris-glycine transfer buffer. Membranes were blocked in 5% (w/v) non-fat dry milk for 1 h at room temperature, followed by protein incubation with primary antibodies including anti-DCX, anti-Nestin (Rabbit, 1:1000; Abcam), anti-BDNF, anti-BMP2, anti-BMP4, anti-Hes5 (Rabbit, 1:500; Abcam), and anti-Notch1 (Rabbit, 1:1,000; CST) at 4°C overnight. Then, the membranes were gently washed with TBST [50 mM Tris-HCl (pH 7.4; Acros Organics BVBA, Geel, Belgium), 150 mM NaCl, and 0.05% Tween 20 (Acros Organics BVBA)] three times and incubated with goat anti-rabbit IgG (H + L) secondary antibodies (1:5,000; LI-COR Biosciences, Lincoln, NE, United States) at room temperature for 1 h. Finally, the membranes were scanned, and the signals of reactive bands were quantified using the Odyssey Infrared Imager (LI-COR). β-Actin was detected as internal control. Gray value levels of the targeted proteins were quantitatively analyzed by the ratio of corresponding proteins to β-actin through ImageJ software (National Institutes of Health, United States). Western blots were duplicated three independent times.

### Statistical Analysis

All results were expressed as means ± standard deviation. Two-way repeated measures analysis of variance (ANOVA) was performed to determine the interaction effect between group and time period on related neurological function, and one-way ANOVA was used to analyze other data followed by Tukey test for multiple comparisons. Differences were considered to have statistical significance at *p* < 0.05. All data were analyzed using SPSS 19.0 (IBM, Armonk, NY, United States).

## Results

### Ginseng-Angelica-Shanseng-Pulvis Extraction Enhanced Sensorimotor Functions and Recognition Memory

**FIGURE 2:**
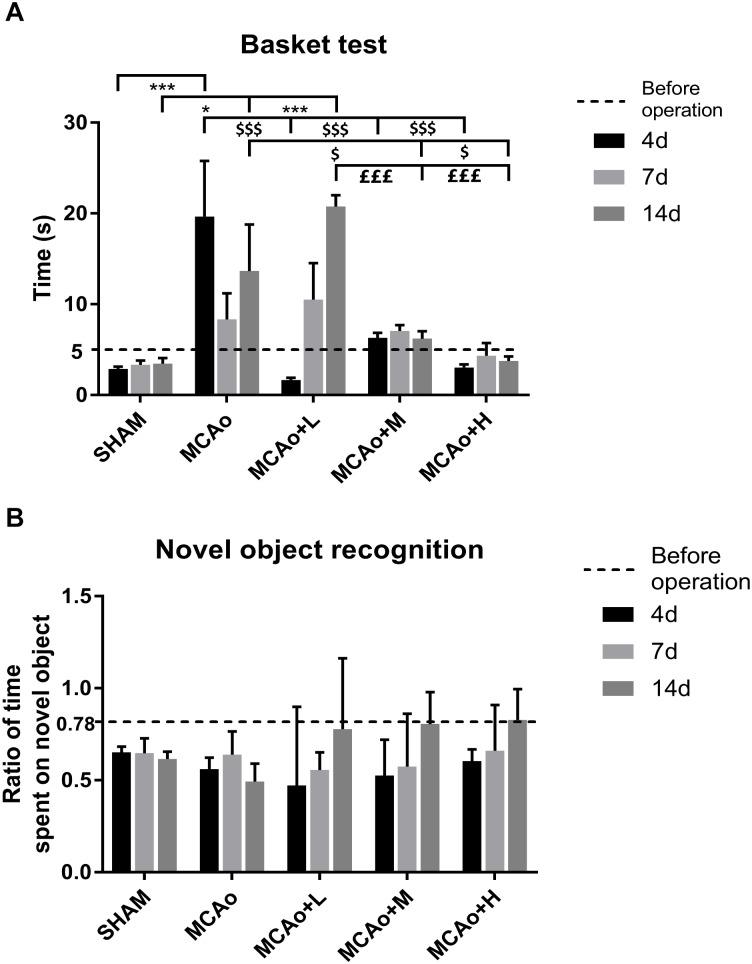
Assessments of basket test and novel object recognition test outcomes. **(A)** The basket test was performed before operation as well as 4, 7, and 14 days after operation in each group. **(B)** Ratio of time spent on novel object was counted at the same time. Data presented as mean ± standard deviation. ^∗^*p* < 0.05, ^∗∗∗^*p* < 0.001 vs. Sham group; ^$^*p* < 0.05, ^$$$^*p* < 0.001 vs. middle cerebral artery occlusion (MCAo) group; ^£££^*p* < 0.001 vs. MCAo + Low-dose Ginseng-Angelica-Shanseng-Pulvis (GASP) (L) group.

In the basket test, a two-way repeated measures ANOVA showed that significant effects for both group and time period regarding neurological function of four MCAo operating groups (group: *F* = 12.12, *p* < 0.001; time period: *F* = 2.709, *p* < 0.001), as well as obvious effects in the factor of group and time period (*F* = 5.655, *p* < 0.001). As shown in Figure 2A, quantitative analysis indicated that the consumed time of the middle- to high-dose group was approximate to that of the Sham group at day 4, and the trend was always maintained at day 14. During the period from 7 to 14 days, it seemed that the rats in the low-dose group consumed slightly longer than those in the MCAo group, but there was no statistical significance.

The adhesive removal test focused on the sensory function of the forelimbs in rats and the coordination of limb movements. Rats’ left forepaw in the MCAo group showed functional deficiency, and the time spent on the right front paw was prolonged to various degrees (Table 2, 4 days: *p* < 0.05 vs. SHAM; 14 days: *p* < 0.01 vs. SHAM). At day 7, the right side in the low-dose group resumed, following the left side recovering to a certain degree at day 14 (14 days left: *p* < 0.05 vs. SHAM). For rats in the middle- and high-dose groups, left anterior paws showed deficient sensory function only at day 4, and the function began to be restored at day 7. Moreover, the high-dose group’s rehabilitated level of sensory function was the best at day 14.

**Table 2:** The result of adhesive removal test.

	Time (s)							
	Before operation		4 days		7 days		14 days	
Groups	Left	Right	Left	Right	Left	Right	Left	Right
SHAM	2.3 ± 0.8	2.6 ± 1.3	2.8 ± 1.0	3.0 ± 0.8	2.5 ± 1.0	2.5 ± 0.5	2.2 ± 0.4	2.2 ± 0.4
MCAo	2.3 ± 0.8	2.6 ± 1.3	No response	6.7 ± 2.3^∗^	No response	3.5 ± 1.7	No response	6.5 ± 3.1^∗∗^
MCAo+L	2.3 ± 0.8	2.6 ± 1.3	No response	No response	No response	6.0 ± 6.2	15.0 ± 15.7^∗^	3.2 ± 3.4
MCAo+M	2.3 ± 0.8	2.6 ± 1.3	No response	5.1 ± 5.1	8.7 ± 6.1^∗^	5.0 ± 2.9	7.3 ± 3.3	5.8 ± 3.9
MCAo+H	2.3 ± 0.8	2.6 ± 1.3	No response	8.8 ± 7.3	7.5 ± 4.9	4.7 ± 3.1	3.5 ± 1.7ccc	4.5 ± 3.9

*Values are mean ± SD. ^∗∗^p < 0.01, ^∗^p < 0.05 vs. SHAM; ^£^p < 0.05 vs. MCAo+L. MCAo, middle cerebral artery occlusion.*

In the novel object recognition test (Figure 2B), at 14 days, post-stroke learning and memory abilities in the three administration groups further improved. Although the data of each group have no statistical difference, it could reflect the ameliorated trend of recognition memory by GASP extraction treatment.

### Ginseng-Angelica-Shanseng-PulvisExtraction Improved Cerebral BloodFlow and Infarction Volume

**FIGURE 3:**
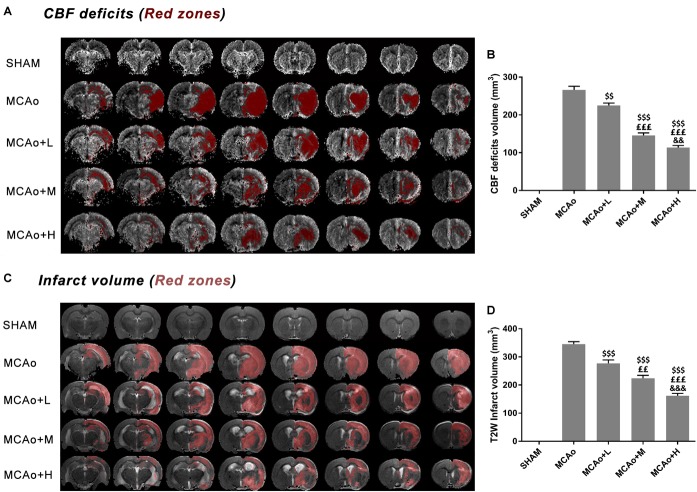
MRI results of regional cerebral blood flow (CBF) deficits and infarction volume evaluation in T2W after 14 days post-MCAo. **(A)** Regional CBF deficit (red zones) images in each group. **(B)** Quantitative analysis of CBF deficits. **(C)** T2W images of infarction volume in different groups. **(D)** Quantitative analysis of cerebral infarction volume. Data presented as mean ± standard deviation. ^$$^*p* < 0.01, ^$$$^*p* < 0.001 vs. MCAo group; ^££^*p* < 0.01, ^£££^*p* < 0.001 vs. MCAo + Low-dose GASP (L) group; ^&&^*p* < 0.01, ^&&&^*p* < 0.001 vs. MCAo + Middle-dose GASP (M) group.

In Figure 3A, DSC images showed a considerable cerebral blood flow (CBF) deficit (red zones) occurring in the ipsilateral hemisphere after stroke. Significantly, the low-, middle-, and high-dose groups ameliorated this status in a dose-dependent manner at day 14 (Figure 3B, *p* < 0.01, MCAo+L vs. MCAo, MCAo+H vs. MCAo+M; *p* < 0.001, MCAo+M and MCAo+H vs. MCAo and MCAo+L). In T2W images (Figure 3C), the red area in the cerebral cortex and striatum represented infarction volume, whose evolution tendency was consistent with CBF, and the high dose of GASP exerted the most significant advantage on regional CBF and infarction volume (Figure 3D, *p* < 0.001 vs. other groups).

### Ginseng-Angelica-Shanseng-PulvisExtraction Increased CellularProliferation in the Subventricular Zone

**FIGURE 4:**
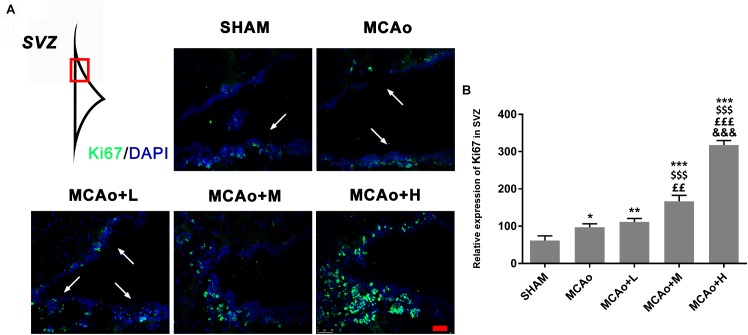
Ginseng-Angelica-Shanseng-Pulvis extraction advanced cell proliferation in the subventricular zone (SVZ) of the ipsilateral hemisphere. **(A)** Immunofluorescence staining with Ki67 (green) was presented (magnification ×200). **(B)** Relative expression of Ki67 in the SVZ was analyzed. Scale bar: 50 μm. Data presented as mean ± standard deviation. ^∗^*p* < 0.05, ^∗∗^*p* < 0.01, ^∗∗∗^*p* < 0.001 vs. Sham group; ^$$$^*p* < 0.001 vs. MCAo group; ^££^*p* < 0.01, ^£££^*p* < 0.001 vs. MCAo + Low-dose GASP (L) group; ^&&&^*p* < 0.001 vs. MCAo + Middle-dose GASP (M) group.

Ki67 is recognized as a marker of cellular proliferation. Immunofluorescence staining showed that cells with green immunofluorescence positive signal (indicated by an arrow) distributed in the SVZ of the ipsilateral hemisphere (Figure 4A). Quantitative analysis indicated that GASP enhanced the expression of Ki67 in a dose-dependent manner; obviously, Ki67 expressions in the middle- and high-dose groups were higher than those in the low-dose and MCAo groups (Figure 4B, *p* < 0.01). The results illustrated that GASP extraction can promote cellular proliferative activity in the SVZ.

### Ginseng-Angelica-Shanseng-PulvisExtraction Enhanced the Expressions ofDoublecortin, Glial Fibrillary AcidicProtein, and Nestin and Brain-DerivedNeurotrophic Factor Secretion

**FIGURE 5:**
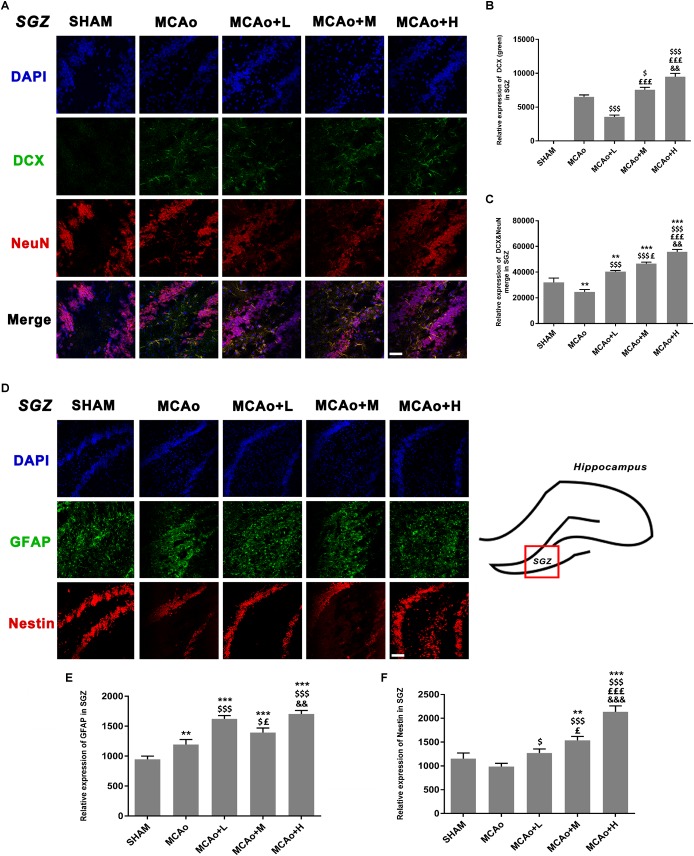
Ginseng-Angelica-Shanseng-Pulvis extraction regulated the expressions of doublecortin, NeuN, glial fibrillary acidic protein, and Nestin in the subgranular zone (SGZ) of the ipsilateral hemisphere. **(A)** Immunofluorescence staining with DCX (green) and NeuN (red) are presented (magnification ×200). Relative expressions of DCX **(B)** and DCX and NeuN merging **(C)** were analyzed. **(D)** The image of immunofluorescence staining with GFAP (green) and Nestin (red) are presented (magnification ×200). Relative expressions of GFAP **(E)** and Nestin **(F)** were analyzed. Scale bar: 50 μm. Data presented as mean ± standard deviation. ^∗∗^*p* < 0.01, ^∗∗∗^*p* < 0.001 vs. Sham group; ^$^*p* < 0.05, ^$$$^*p* < 0.001 vs. MCAo group; ^£^*p* < 0.05, ^£££^*p* < 0.001 vs. MCAo + Low-dose GASP (L) group; ^&&^*p* < 0.01, ^&&&^*p* < 0.001 vs. MCAo + Middle-dose GASP (M) group.

**FIGURE 6:**
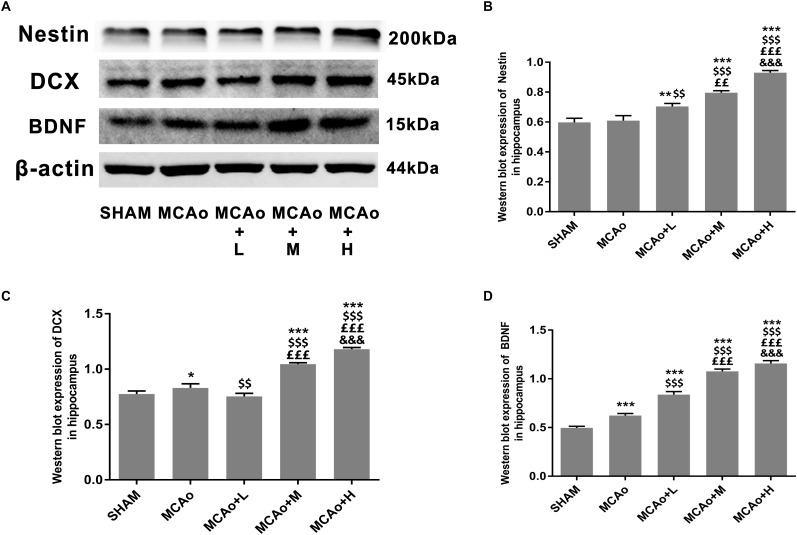
Ginseng-Angelica-Shanseng-Pulvis extraction regulated hippocampal Nestin, DCX, and brain-derived neurotrophic factor (BDNF) expressions. **(A)** Representative Western blot results for Nestin, DCX, and BDNF in each group’s hippocampus and quantitative analysis for Nestin **(B)**, DCX **(C)**, and BDNF **(D)**. Data presented as mean ± standard deviation.^∗^*p* < 0.05, ^∗∗^*p* < 0.01, ^∗∗∗^*p* < 0.001 vs. Sham group; ^$$^*p* < 0.01, ^$$$^*p* < 0.001 vs. MCAo group; ^££^*p* < 0.01, ^£££^*p* < 0.001 vs. MCAo + Low-dose GASP (L) group; ^&&&^*p* < 0.001 vs. MCAo + Middle-dose GASP (M) group.

As shown in Figure 5A, cells with green DCX immunofluorescence positive signal distributed in the SGZ. Quantitative analysis of both immunofluorescence staining (Figure 5B) and Western blotting (Figure 6A,C) indicated that the expression of DCX in the middle- and high-dose groups was significantly higher than that of the low-dose and MCAo groups. Interestingly, immunofluorescence staining suggested that the low-dose group decreased DCX expression compared with the MCAo group (*p* < 0.001), and the trend of DCX expression was also presented by Western blot assay. However, the double-staining result indicated that DCX expression in NeuN-positive cells in the low-dose group was obviously higher than that in the MCAo group (Figure 5C, *p* < 0.001), suggesting that the low dose of GASP contributed to promoting neuronal immaturity. Additionally, Western blot results showed that stress of stroke also stimulated more BDNF secretion in the hippocampus of the ipsilateral hemisphere, and the increased trend of BDNF secretion was dose-dependent after administration of GASP at day 14 (Figure 6A,D; *p* < 0.001).

As shown in Figure 5D, cells with green GFAP^+^ immunofluorescence signal are located in the SGZ. Quantitative analysis showed that the expression of GFAP in the low-dose group was higher than that in the middle-dose group (*p* < 0.05), and although GFAP expression in the high-dose group was the highest (*p* < 0.001 vs. SHAM, MCAo; *p* < 0.01 vs. MCAo+M), there was no statistical significance between the low- and the high-dose groups (Figure 5E). Qualitative analysis of Nestin expression in three administration groups showed an increasing trend in a dose-dependent manner whether in immunofluorescence staining (Figure 5F) or in Western blot (Figure 6B). These might be explained by the fact that the efficacies of different dosage of GASP on NSCs were distinct: a low dose perhaps contributes to NSC differentiation into glia cells in the SGZ, while a high dose not only enhanced NSCs along with radial glia cellular differentiation, but also advanced NSCs to differentiate into non-radial type 2 cells. Interestingly, the action of middle dose on NSC differentiation appeared in the transformed process from radial glia cells to non-radial type 2 cells.

### Ginseng-Angelica-Shanseng-Pulvis Extraction Regulated Bone Morphogenetic Protein 2/4 and Notch1/Hairy and Enhancer of Split 5 Signal Pathways in the Hippocampus

**FIGURE 7:**
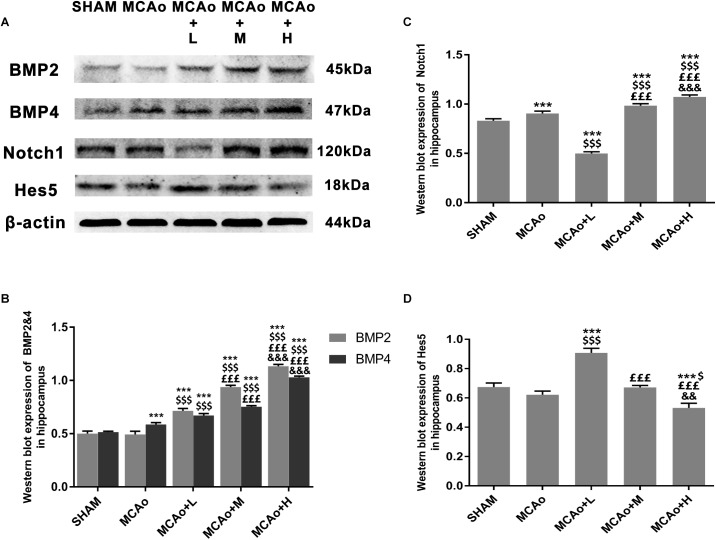
Ginseng-Angelica-Shanseng-Pulvis extraction manipulated BMP2/4 and Notch1/Hes5 pathways in the hippocampus. **(A)** Representative Western blot belts for BMP2, BMP4, Notch1, and Hes5 in each group. **(B–D)** Quantitative analysis for the above proteins. Data presented as mean ± standard deviation. ^∗∗∗^*p* < 0.001 vs. Sham group; ^$^*p* < 0.05, ^$$$^*p* < 0.001 vs. MCAo group; ^£££^*p* < 0.001 vs. MCAo + Low-dose GASP (L) group; ^&&^*p* < 0.01, ^&&&^*p* < 0.001 vs. MCAo + Middle-dose GASP (M) group.

In order to clarify mechanisms of GASP extraction on neurogenesis, we investigated key signal regulation pathways. As shown in Figure 7A,B, GASP enhanced the expressions of BMP2 and BMP4 in a dose-dependent manner compared with the MCAo group (*p* < 0.001). Surprisingly, the low-dose group inhibited Notch1 and increased Hes5 expressions (Figure 7A,C,D; *p* < 0.001), together with its high expression of GFAP in the SGZ (Figure 5D,E), indicating that astrocyte-like differentiation was related to suppressed Notch1 and enhanced Hes5 signals, which was consistent with Guo et al. (2009) results. In contrast, the trend was reversed in the middle- and high-dose groups. The high-dose group promoted Notch1 expression and suppressed Hes5 expression more significantly compared with the middle-dose group (*p* < 0.01).

## Discussion

**FIGURE 8:**
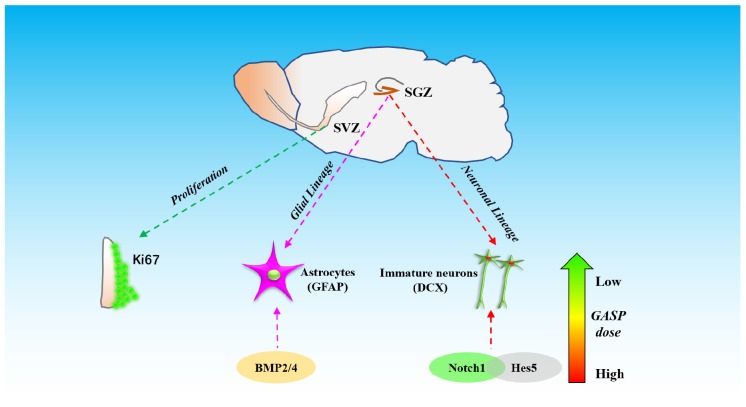
The schematic diagram of different effects of three dosages of GASP on neurogenesis in SGZ and SVZ after ischemic stroke.

These data demonstrate that GASP increases neurogenesis by increasing the proliferative activity of cells in the SVZ and the potential of cells to differentiate in the post-ischemic SGZ. This is associated with significant improvements to regional CBF, decreases in cerebral infarction volume, and an enhancement of BDNF secretion in the hippocampus. Improvements to sensorimotor and recognition memory were also observed. These may be related to the activation of BMP2 and BMP4 and regulation of Notch1/Hes5 pathways in the hippocampal zone of the ipsilateral hemisphere (Figure 8).

Accumulating evidence suggests that stress of stroke can stimulate cellular proliferation in the SVZ and neurogenesis (Zhang et al., 2007; Parent et al., 2010), and SVZ cellular proliferative markers gradually increase and peak at day 14 in rats with stroke (Zhang et al., 2001). Newly matured neurons found in the ischemic striatum and cortex may be related to their neuronal migration from the SVZ (Thored et al., 2006; Yamashita et al., 2006). We used Ki67, an endogenous proliferative marker, to assess SVZ cellular proliferative ability at day 14 post-MCAo. The result of immunofluorescence staining showed that Ki67 expression in the SVZ began to increase after stroke, which indicated that cellular proliferative ability was activated, and this efficacy was further amplified by the administration of GASP in a dose-dependent manner.

Nestin has been widely accepted as a primary marker of progenitor/stem cell populations (Halbach, 2011). Our Western blot and immunofluorescence staining results showed that the administration of GASP advanced Nestin expression to grow steadily with the dose-dependent trend, suggesting that GASP could obviously increase the number of progenitor/stem cell populations in the hippocampus. The progenitor/stem cells usually differentiate into radial glia cells, which express the astrocytic marker, e.g., GFAP, and/or non-radial type 2 precursor cells, which express DCX (a classic immature neuronal marker) (Mu et al., 2010). In the present study, both Western blot and immunofluorescence staining results showed that the expressions of DCX and DCX merged with NeuN (a mature neuron marker) in the middle- and high-dose GASP groups were significantly higher, showing that the dosage scope boosted NSCs/progenitor cells to differentiate into immature neurons and promoted their maturity. Interestingly, the low-dose group decreased DCX expression, but its expression of DCX-merged NeuN was higher than that of the MCAo group, indicating that the treatment group did not facilitate the precursor cell lineage to differentiate into immature neurons and only slightly elevate maturity of DCX^+^ cells. Moreover, we are surprised to find that in the SGZ, GFAP immunofluorescence expression in the low-dose group was higher than that in the middle-dose group and approximate to the high-dose group, suggesting that low-dose GASP increased the NSCs’ differentiated ability along with radial glia cell lineage.

Increasing stroke-induced neurogenesis contributes to the improvement of neurological functional outcomes (Leker et al., 2007). Ginsenoside Rg1 enhances ischemia-induced cell proliferation in the dentate gyrus and improves neurological function (Gao et al., 2010). Our previous study also found that combination treatment of SF and BP derived from *A. sinensis* notably advanced neurogenesis in ischemic boundary zones and ameliorated neurological function and infarction volume (Zhang et al., 2016). Meanwhile, Trans-cinnamaldehyde, an essential oil in cinnamon powder, also exerts neuroprotective effects and reduces infarction against ischemic stroke (Chen et al., 2016). Our herbal extraction contains these active ingredients, and we have demonstrated that its use improved sensorimotor and object recognition at both high and middle concentrations. We hypothesize that these effects are associated with the promotion of neuron-like differentiation in the hippocampus. Although low-dose GASP enhances astrocyte-like differentiation, it appears to have a negative effect on neurological functional amelioration after stroke.

Additionally, BDNF exerts important actions in protecting brain tissue against injury as well as facilitating neuronal plasticity and neurogenesis. In the hippocampus, it plays a crucial role in learning and memory (Yamada and Nabeshima, 2003). A previous study discovered that exogenous administration of BDNF by intrahippocampal infusion could increase neurogenesis in adult rats (Scharfman et al., 2005). Therefore, BDNF plays an essential role in promoting neurogenesis, and our study shows that its effects are mediated in a dose-dependent manner. Combining the ameliorated sensorimotor and recognition memory in the present study, BDNF further aims to improve behavioral assessment by GASP.

Notch1 and BMP2/4 are two important genes regulating NSC/progenitor cell differentiation signal pathways. Overexpression of Notch1 promotes proliferation of neural progenitor cells (Breunig et al., 2007). It suggests that elimination of Notch1 from inducible Nestin^+^ mice decreases NSC number and neuro-like differentiation in the SGZ (Ables et al., 2010). Through the silencing of Hes5, a repressor for tissue-specific gene transcription and the target gene of Notch, proneurogenic Bcl6 recruits SIRT1 to advance neurogenesis (Tiberi et al., 2012). Further research reports that in Hes1-, Hes3-, and Hes5-deficient embryos, neuroepithelial cells are not properly maintained, and radial glial cells are accelerated to differentiate into neurons (Hatakeyama et al., 2004). It suggests that suppression of Hes-5 contributes to neuro-like differentiation. Presently, we demonstrated that significant upregulation of Notch1 and downregulation of Hes5 in the high-dose GASP group resulted in the promotion of NSC/progenitor cell division into neuron-like cells. In turn, low-dose GASP decreased the level of Notch1 but reversed its target Hes5 in the hippocampus. Guo and colleagues found that chronic unpredicted mild stress causes decreasing number of neurogenically differentiating cells in the SGZ of adult ischemic rats after 28 days, but it accelerated astrocyte-like cell differentiation by suppressing Notch1 and enhancing Hes1 and Hes5 expressions (Guo et al., 2009). Therefore, we think that numerous GFAP^+^ cells of the SGZ in the low-dose group may be attributed to inhibited Notch1 and activated Hes5 signals.

The BMP signaling pathway also plays a key regulatory role in different stages of central nervous system development. During embryonic neural development, knockdown of BMP2/4/7 in *Xenopus* embryos caused ubiquitous neural induction throughout the ectoderm (Reversade and Robertis, 2005), and different BMP receptors had varying roles in controlling neural precursor cell production and fate (Panchision et al., 2001). BMP2 and BMP4 can promote adult neural precursor cells differentiating into astrocyte (Gross et al., 1996). Our results indicated that GASP upregulated both BMP2 and BMP4 in a dose-dependent manner in the SGZ, which can be used to explain why high dose induced obvious astrocyte-like cell differentiation. Although BMP2/4 expression in the low-dose group was lower than those in the middle- and high-dose groups, combined with downregulation of Notch1 signal, it boosted NSC/progenitor cell differentiation along with astrocytic direction. Therefore, different dosages of GASP determine cell division fate in the SGZ after ischemic stroke. In the present study, there are some limitations that need to be elucidated in future studies. For example, we did not test the differentiated abilities and mechanisms of neuroblasts derived from the SVZ, so we will focus on research following experiments.

In summary, traditionally, we demonstrate a GASP extract in advancing neurogenesis. BMP2/4 and Notch1/Hes5 pathways are involved in its regulation mechanisms. Due to its beneficial efficacy, GASP shows considerable promise as an alternative therapeutic agent for ischemic stroke.

## Ethics Statement

This study was carried out in accordance with the principles of the NIH Guide for the Care and Use of Laboratory Animals (National Institutes of Health Publication No. 80-23, revised 1996) and recommendations of Animals in Neuroscience Research by the Society for Neuroscience, Institutional Animal Care and Use Committee of Macau University of Science and Technology. The protocol was approved by the Institutional Animal Care and Use Committee of Macau University of Science and Technology.

## Author Contributions

YZ designed the research. BL, ZX, CL, and YL completed by grouping, modeling, and administration of experimental animals. QZ and CK finished MRI detection and analysis. BL wrote the manuscript. Thanks to YZ, XG, XC, and YX for their valuable suggestions in the revision and improvement of the manuscript.

## Conflict of Interest Statement

The authors declare that the research was conducted in the absence of any commercial or financial relationships that could be construed as a potential conflict of interest.
